# A Buddy When You Need One: Buddy Balloon-Assisted Bailout for Failed Valve Crossing During Transfemoral Transcatheter Aortic Valve Implantation

**DOI:** 10.7759/cureus.111746

**Published:** 2026-06-29

**Authors:** Vasileios Leivaditis, Sofien Ayed, Roman Gottardi, Athanasios Papatriantafyllou, Robert Bernat, Manfred Dahm, Burghard Schumacher

**Affiliations:** 1 Department of Cardiothoracic and Vascular Surgery, Westpfalz-Klinikum, Kaiserslautern, DEU; 2 Department of Internal Medicine 2, Westpfalz-Klinikum, Kaiserslautern, DEU

**Keywords:** bailout strategy, balloon-expandable valve, buddy-balloon technique, severe aortic stenosis, transcatheter aortic valve implantation (tavi), valve crossing failure, valvular calcification

## Abstract

Failure of transcatheter heart valve advancement across a severely calcified native aortic valve is an uncommon but potentially procedure-threatening complication during transcatheter aortic valve implantation (TAVI). Various bailout techniques have been described; however, published experience with the buddy-balloon technique remains limited. A 77-year-old man with symptomatic severe calcific aortic stenosis (mean gradient 90 mmHg, valve area 0.5 cm²) was referred for transfemoral TAVI. Preprocedural imaging demonstrated extreme valvular calcification with favorable iliofemoral access. Following successful valve crossing, stiff guidewire placement, and balloon aortic valvuloplasty, advancement of a 26-mm balloon-expandable transcatheter heart valve remained impossible despite multiple conventional maneuvers, including flexion and optimization of coaxial alignment. A bailout buddy-balloon technique was therefore performed through secondary femoral arterial access. Repeat balloon inflation across the native valve temporarily modified the geometry of the heavily calcified valve complex, enabling successful passage and deployment of the prosthesis. Final angiography and echocardiography demonstrated an excellent procedural result, with only mild paravalvular regurgitation and no periprocedural complications. Severe valvular calcification remains an important cause of procedural complexity during TAVI. This case illustrates that the buddy-balloon technique may represent a feasible bailout strategy when standard methods fail to facilitate valve crossing. Familiarity with this maneuver may provide operators with an additional bailout option when confronted with challenging anatomy during transfemoral TAVI.

## Introduction

Transcatheter aortic valve implantation (TAVI) has become an established treatment for patients with severe symptomatic aortic stenosis and is currently routinely performed through a transfemoral approach whenever anatomically feasible [1-3]. Advances in valve design, delivery systems, and preprocedural computed tomography planning have significantly improved procedural success and reduced complications. Nevertheless, challenging anatomical substrates, particularly severe leaflet and annular calcification, may still create important technical obstacles during valve delivery and deployment [4-6].

Among the procedural challenges encountered during TAVI, failure of the delivery system to cross the native aortic valve remains an uncommon but potentially procedure-threatening event. Excessive valvular calcification, asymmetric calcium distribution, extreme valve narrowing, unfavorable root geometry, horizontal aortas, and inadequate coaxial alignment may prevent advancement of the prosthesis despite successful guidewire positioning and predilatation [7-9]. In such situations, repeated attempts at forceful advancement may increase the risk of annular injury, embolization of calcific debris, vascular complications, or procedural failure, emphasizing the need for alternative bailout strategies [4,7,10].

Several adjunctive techniques have been described to facilitate crossing of severely stenotic and calcified aortic valves, including buddy-wire support, repeat balloon aortic valvuloplasty, balloon-cushion techniques, snare-assisted delivery, and buddy-balloon maneuvers [8-12]. The buddy-balloon technique, initially described by Sheiban I et al. and subsequently reported by Balkin J et al., uses simultaneous balloon inflation across the native valve to transiently modify valve geometry and create sufficient space for advancement of the transcatheter valve system [11-13]. Although the technique is simple and readily applicable through standard transfemoral access, published experience remains limited to isolated case reports and small case series.

This report describes a patient with critical, severely calcified aortic stenosis in whom conventional advancement of a balloon-expandable transcatheter heart valve was impossible despite successful valve crossing, stiff-wire support, and prior balloon valvuloplasty. Successful device delivery and implantation were ultimately achieved using a buddy-balloon bailout technique, thereby avoiding procedural abandonment or conversion to an alternative access strategy.

## Case presentation

A 77-year-old man was referred to our Heart Team for TAVI because of severe symptomatic aortic valve disease. His medical history was notable for persistent atrial fibrillation treated with apixaban, biatrial enlargement, mild mitral regurgitation, moderate tricuspid regurgitation, and prostate carcinoma under oncological follow-up. Coronary angiography performed prior to the intervention demonstrated no significant coronary artery disease.

The patient reported progressive exertional dyspnea corresponding to New York Heart Association (NYHA) functional class II-III. Physical examination revealed a loud systolic ejection murmur radiating to both carotid arteries and mild peripheral edema.

**Figure 1:**
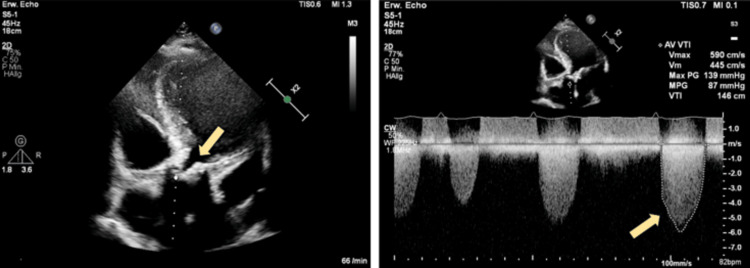
Preprocedural echocardiographic assessment of critical calcific aortic stenosis. (A) Transthoracic echocardiography demonstrating a severely calcified native aortic valve with markedly restricted leaflet mobility and virtually absent leaflet separation (arrow). (B) Continuous-wave Doppler interrogation across the aortic valve showing severe transvalvular obstruction, with a peak velocity of approximately 5.9 m/s and a mean gradient of 90 mmHg (arrow), consistent with critical aortic stenosis.

Transthoracic echocardiography demonstrated a severely calcified native aortic valve with virtually absent leaflet separation. Hemodynamic assessment revealed critical aortic stenosis, with a mean transvalvular gradient of 90 mmHg and an aortic valve area of 0.5 cm² (Figure 1). Mild aortic regurgitation was also present. Left ventricular systolic function was preserved, with concentric left ventricular hypertrophy and no regional wall motion abnormalities.

**Figure 2:**
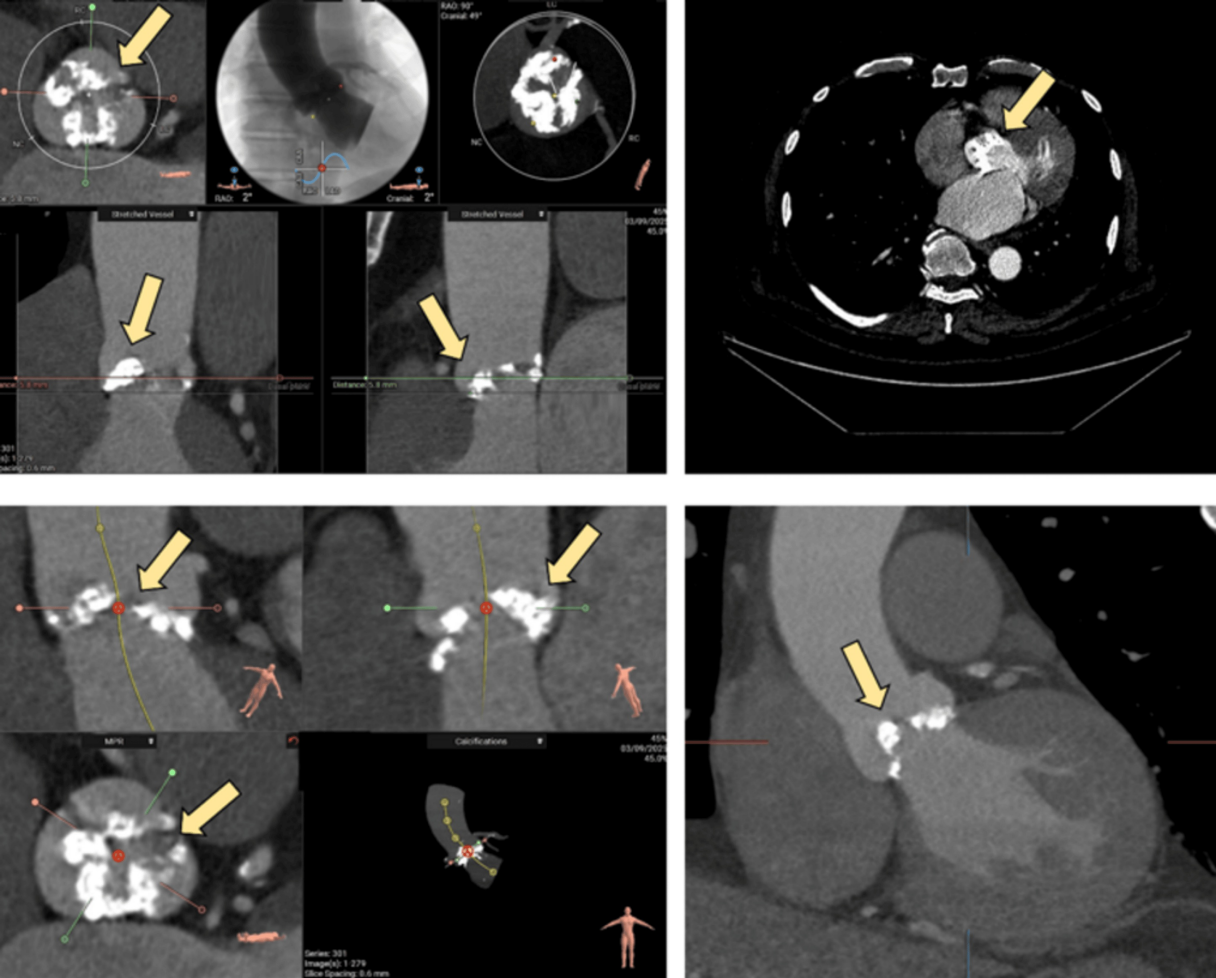
Preprocedural computed tomography demonstrating extensive aortic valve calcification. Multiplanar reconstruction images of the aortic root and aortic valve complex show massive leaflet and annular calcification (arrows). The extensive and asymmetric calcium burden resulted in a critically stenotic valve orifice and represented the principal anatomical challenge for transcatheter valve delivery.

Preprocedural computed tomography confirmed extensive calcification of the aortic valve complex, involving both the leaflets and annular region (Figure 2). The iliofemoral vessels were of adequate caliber, without significant obstructive peripheral arterial disease, supporting a transfemoral approach. Following multidisciplinary discussion, the Heart Team recommended transfemoral TAVI.

The procedure was performed in a hybrid operating room under local anesthesia with conscious sedation. Ultrasound-guided vascular access was obtained through the right common femoral artery (14-French sheath), left common femoral artery (6-French sheath), and left femoral vein (5-French sheath). A temporary pacing lead was positioned in the right ventricular apex via femoral venous access. A pigtail catheter was advanced to the aortic root for angiographic guidance.

Crossing the native valve proved challenging because of the extreme degree of calcification and critical stenosis. After several attempts, the valve was crossed using a straight Terumo wire and JR4 catheter. Subsequently, a Confida stiff guidewire (Medtronic, Minneapolis, MN, USA) was positioned in the left ventricle. Balloon aortic valvuloplasty was performed using a 22-mm balloon under rapid ventricular pacing.

Based on annular measurements, a 26-mm balloon-expandable Edwards SAPIEN 3 Ultra valve (Edwards Lifesciences, Irvine, CA, USA) was selected. The valve delivery system was advanced through the transfemoral route without difficulty to the ascending aorta. However, despite appropriate positioning of the stiff guidewire and prior balloon predilatation, the transcatheter heart valve could not be advanced across the native aortic valve. Multiple conventional maneuvers were attempted, including repeated adjustments of catheter orientation, flexion of the delivery system, and optimization of coaxial alignment. Nevertheless, the prosthesis repeatedly failed to cross the heavily calcified annulus.

**Figure 3:**
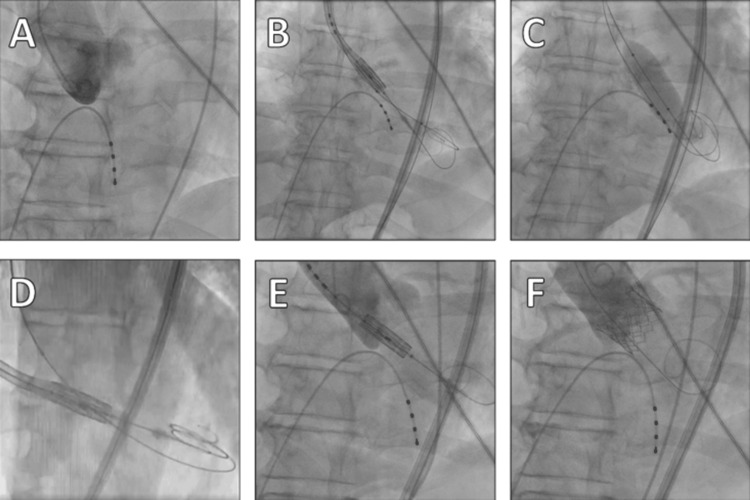
Buddy-balloon-assisted facilitation of valve crossing during transfemoral TAVI. Stepwise application of the buddy-balloon technique: (A) Initial aortography demonstrating severe aortic stenosis. (B) Failure of the Edwards SAPIEN 3 Ultra delivery system to cross the heavily calcified native valve despite predilatation and optimal guidewire support. (C) Re-crossing of the valve through secondary arterial access and positioning of a second stiff guidewire in the left ventricle. (D) Inflation of a 22-mm buddy balloon across the native valve during rapid ventricular pacing. (E) Successful advancement of the transcatheter heart valve across the aortic annulus, facilitated by temporary modification of valve geometry. (F) Final deployment of the balloon-expandable prosthesis in the optimal annular position. TAVI: Transcatheter aortic valve implantation.

Given the inability to advance the valve despite standard techniques, a bailout buddy-balloon strategy was adopted. The left femoral arterial access was upsized to an 11-French sheath. The native valve was re-crossed using an AL1 catheter and Terumo guidewire, allowing placement of a second Confida stiff guidewire within the left ventricle. A second balloon aortic valvuloplasty was then performed using a 22-mm balloon during rapid ventricular pacing while maintaining the primary valve delivery system immediately proximal to the aortic annulus (Figure 3).

**Figure 4:**
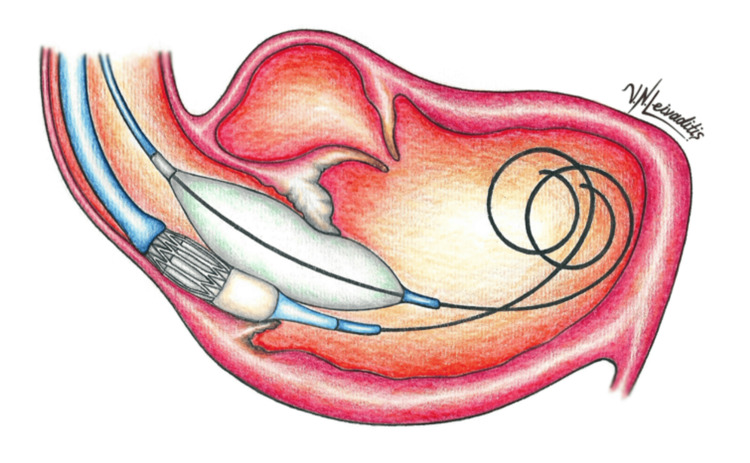
Schematic illustration of the buddy-balloon technique. Repeat balloon valvuloplasty temporarily increases valve compliance and modifies the geometry of the severely calcified native valve. During balloon deflation, the balloon may additionally act as a mechanical “shoehorn,” facilitating advancement of the transcatheter heart valve delivery system across the stenotic valve. Note: Original hand-drawn illustration created by Leivaditis V (first author); no digital or generative AI tools or external sources were used.

Following repeat balloon valvuloplasty, the transcatheter heart valve was advanced during balloon deflation. The simultaneous placement of the delivery device and the assisting buddy balloon is schematically represented in Figure 4. The combination of transient valve expansion and the mechanical “shoehorn” effect created by the deflating balloon facilitated successful crossing of the severely calcified native valve. During this maneuver, the Edwards SAPIEN 3 Ultra delivery system successfully crossed the native valve and was positioned within the annulus.

Following confirmation of optimal positioning by fluoroscopy and aortography, the valve was deployed under rapid ventricular pacing. Final angiography demonstrated excellent prosthesis position with only trivial residual aortic regurgitation. No coronary obstruction, annular injury, conduction disturbance, or vascular complications occurred.

Postprocedural transthoracic echocardiography confirmed a well-functioning transcatheter heart valve with a mean transprosthetic gradient of 18 mmHg and only two minimal paravalvular jets, corresponding to mild (grade I) paravalvular regurgitation. Left ventricular systolic function remained preserved, and no pericardial effusion was detected.

The patient’s postprocedural course was uneventful. Telemetry showed persistent atrial fibrillation without new conduction abnormalities. He was discharged home in stable clinical condition on postprocedural day four, with continuation of oral anticoagulation therapy and scheduled follow-up in the structural heart clinic. The chronological sequence of diagnostic evaluation, procedural management, and clinical outcomes is summarized in Table 1.

**Table 1:** Timeline of the patient’s clinical presentation, diagnostic workup, procedural course, and postprocedural outcome. Summary of key events from initial presentation with symptomatic severe aortic stenosis through preprocedural assessment, transcatheter aortic valve implantation, buddy-balloon bailout strategy, and clinical follow-up. AVA: Aortic valve area; CT-TAVI: Computed tomography for transcatheter aortic valve implantation; NYHA: New York Heart Association; PVL: Paravalvular leak; TAVI: Transcatheter aortic valve implantation; TTE: Transthoracic echocardiography.

Timepoint	Event
Pre-admission	Progressive exertional dyspnea (NYHA class II–III)
TTE	Critical aortic stenosis (AVA 0.5 cm², mean gradient 90 mmHg)
Coronary angiography	No significant coronary artery disease
CT-TAVI	Severe leaflet and annular calcification; suitable transfemoral access
Heart Team	Decision for transfemoral TAVI
Procedure	Balloon valvuloplasty followed by failed valve crossing
Bailout	Buddy-balloon technique performed
Outcome	Successful implantation of a 26-mm SAPIEN 3 Ultra valve
Post-procedure TTE	Mean gradient 18 mmHg; mild PVL
Discharge	Uneventful recovery; discharge on day 4

## Discussion

This case illustrates a rare but important technical challenge during TAVI: the inability to advance a balloon-expandable transcatheter heart valve across an extremely calcified native aortic valve despite successful valve crossing, adequate guidewire support, and prior balloon valvuloplasty. Although contemporary TAVI systems have substantially improved deliverability and procedural success rates, severe valvular calcification continues to represent a major anatomical obstacle that may complicate device advancement and deployment [4-6,14,15].

Extensive leaflet and annular calcification can alter valve geometry, reduce leaflet compliance, and create a rigid mechanical barrier to prosthesis passage. In addition, a heavy, asymmetric calcium burden may impair coaxial alignment of the delivery system and increase friction between the transcatheter valve frame and the native valve complex [6,14,16]. Such anatomical features have been associated with increased procedural complexity, a greater need for adjunctive maneuvers, and higher risks of complications, including annular injury, prosthesis malposition, paravalvular regurgitation, and embolic events [6,16-18]. In the present case, preprocedural imaging demonstrated extreme valvular calcification, while echocardiography revealed critical stenosis with virtually absent leaflet separation, explaining the substantial resistance encountered during valve advancement. The principal bailout strategies described for failed valve crossing during TAVI, together with their mechanisms, advantages, and limitations, are summarized in Table 2.

**Table 2:** Bailout strategies for failed transcatheter heart valve crossing during TAVI. Overview of the principal techniques described to facilitate transcatheter heart valve delivery when conventional advancement across the native valve fails, including their proposed mechanisms, advantages, limitations, and representative references from the published literature. TAVI: Transcatheter aortic valve implantation.

Technique	Mechanism	Advantages	Limitations	Key references
Repeat balloon aortic valvuloplasty	Further enlarges the stenotic valve orifice and disrupts calcific restraint	Simple, familiar, and widely available	May remain ineffective in extreme calcification; potential embolic risk	[7,10]
Buddy-wire technique	Additional left ventricular guidewire increases support and improves device tracking	Easy to perform; no additional large devices required	Limited efficacy when severe calcification is the primary obstacle	[19]
Balloon-nudge technique	Balloon inflation modifies device trajectory and improves coaxial alignment	Useful for non-coaxial valve delivery systems	Less effective when crossing failure is due to critical calcific obstruction	[8]
Long-sheath support	Improves pushability and delivery-system support	Helpful in tortuous anatomy or cases of inadequate support	Requires additional equipment and vascular manipulation	[20]
Snare-assisted delivery	Improves coaxial alignment and facilitates controlled advancement	Effective in selected anatomies	Increased procedural complexity and need for additional equipment	[21]
Buddy-balloon technique	Temporary balloon inflation alters valve geometry and separates calcified leaflets, facilitating prosthesis crossing	Simple and rapid; uses standard TAVI equipment; particularly useful in heavily calcified valves	Limited published experience; requires secondary arterial access	[9,11-13,22]
Alternative access routes (transapical, transaortic, transsubclavian, transcaval)	Bypass unfavorable anatomy or delivery constraints	May rescue otherwise unsuccessful procedures	Increased invasiveness and procedural complexity	[23-25,26]

Failure to cross the native valve after successful guidewire placement is uncommon but remains a recognized challenge in structural heart interventions. Several bailout techniques have been described to facilitate prosthesis delivery in this setting. These include optimization of wire position within the left ventricle, use of additional stiff “buddy” guidewires, repeat balloon aortic valvuloplasty, long-sheath support, snare-assisted delivery, balloon-cushion techniques, and alternative access routes [7-12,19-21]. Selection among these strategies depends on the underlying mechanism preventing valve crossing, operator experience, and procedural risk considerations.

The buddy-balloon technique represents one of the most elegant and readily available solutions for failed valve crossing in heavily calcified anatomy. Originally described by Sheiban I et al. [11] and subsequently reported by Balkin J et al. [12] and Okuyama K et al. [13], the technique uses simultaneous balloon inflation across the stenotic valve through secondary arterial access while the transcatheter valve delivery system is positioned immediately proximal to the annulus [11-13]. Inflation of the balloon temporarily alters the geometry of the calcified valve apparatus, separates rigid leaflet calcifications, and creates a transient enlargement of the effective orifice area that facilitates advancement of the prosthesis [12,13]. Interestingly, the mechanism by which the buddy-balloon technique facilitates valve crossing may differ according to the manner in which it is applied. In the original description by Sheiban I et al. [11], the balloon was inflated at low pressure and acted as a “percutaneous shoehorn,” creating a smooth ramp that altered the geometry of the crossing point and allowed the prosthetic valve to slide across the stenotic native valve. In contrast, the approach followed in this case involved repeat balloon aortic valvuloplasty using a 22-mm balloon at standard inflation pressure. This maneuver likely provided an additional increase in valve compliance and effective orifice area, while advancement of the transcatheter heart valve during balloon deflation may have reproduced the shoehorn effect described by Sheiban I et al. It is likely that successful crossing of the severely calcified valve resulted from a combination of these mechanisms. A schematic representation of the proposed mechanism is shown in Figure 4.

Computed tomography analyses have further suggested that this maneuver modifies the interaction between the delivery system and heavily calcified commissural structures, thereby reducing resistance during crossing [13]. To our knowledge, published experience with buddy-balloon-assisted TAVI remains limited to isolated case reports and small case series, making the present report a further contribution to the evidence supporting this technique.

In this case, conventional maneuvers, including repeated flexion and angulation of the delivery system, failed to achieve valve passage. The decision was therefore made to perform repeat balloon valvuloplasty using a buddy-balloon strategy through a second femoral arterial access. This approach immediately enabled successful advancement of the Edwards SAPIEN 3 Ultra valve across the native annulus without the need for procedural abandonment or conversion to an alternative access route. Similar successful experiences have been reported in isolated case reports and small series, supporting the reproducibility and practicality of this technique in carefully selected patients [9,12,13,22].

Key features that may alert operators to this scenario include extensive leaflet and annular calcification on preprocedural imaging, critical valve narrowing, inability to advance the transcatheter heart valve despite successful guidewire positioning and balloon predilatation, and persistence of crossing failure despite conventional delivery maneuvers and adequate guidewire support.

An important advantage of the buddy-balloon technique is that it can be performed rapidly using equipment routinely available during TAVI procedures. Unlike alternative access strategies such as transapical, transaortic, transsubclavian, or transcaval approaches, the technique avoids additional vascular trauma and procedural complexity [23-25]. Furthermore, compared with aggressive forceful advancement of the prosthesis, buddy-balloon assistance may reduce the risk of annular rupture, valve embolization, or damage to the delivery system by creating a controlled modification of valve geometry [6,17,18].

The present case also highlights the importance of meticulous preprocedural imaging and procedural planning in patients with extreme calcific aortic valve disease. Contemporary multidetector computed tomography remains essential for assessment of annular dimensions, calcium distribution, vascular access, and prediction of procedural challenges [13,26-29]. Nevertheless, even comprehensive preprocedural imaging cannot fully predict all mechanical difficulties encountered during valve delivery. Therefore, familiarity with bailout techniques remains an important component of the structural interventionalist’s armamentarium.

Although experience with buddy-balloon-assisted TAVI remains limited, the available literature suggests that it is a safe and effective strategy when standard methods fail. As TAVI continues to expand to increasingly complex anatomical subsets, awareness of such procedural solutions may help operators avoid procedural abandonment and maintain the advantages of a transfemoral approach. Larger procedural registries and multicenter experiences would be valuable to better define indications, technical considerations, and outcomes associated with this technique.

## Conclusions

This case highlights the buddy-balloon technique as a potentially useful bailout strategy for failed valve crossing during transfemoral TAVI. In the setting of extreme valvular calcification, temporary modification of valve geometry enabled successful delivery of a balloon-expandable prosthesis and avoided the need for alternative access or procedural abandonment. Awareness of this technique may provide operators with an additional bailout option when confronted with failed valve crossing during transfemoral TAVI. Further experience is required to better define its role, safety, and reproducibility.

This case offers several practical learning points. Severe aortic valve calcification may prevent advancement of a transcatheter heart valve despite successful valve crossing and predilatation. Standard maneuvers, including flexion, coaxial optimization, and repeat predilatation, may occasionally be insufficient. The buddy-balloon technique can temporarily alter valve geometry and facilitate prosthesis crossing in heavily calcified anatomy. Familiarity with bailout strategies may help avoid procedural abandonment and preserve transfemoral access.
